# Primary transitional cell carcinoma of the fallopian tube: A case report and literature review

**DOI:** 10.1097/MD.0000000000020499

**Published:** 2020-05-29

**Authors:** Dong-Hyun Lee, Dong-Hyu Cho, Kyoung Min Kim, Chang-Yeol Yim, Na-Ri Lee

**Affiliations:** a Department of Obstetrics and Gynecology, Jeonbuk National University Hospital-Jeonbuk National University Medical School; b Research Institute of Clinical Medicine of Jeonbuk National University-Biomedical Research Institute of Jeonbuk National University Hospital; c Department of Pathology; d Division of Hematology and oncology, Department of Internal Medicine, Jeonbuk National University Hospital-Jeonbuk National University Medical School, Jeonju, Republic of Korea.

**Keywords:** fallopian tube, transitional cell carcinoma

## Abstract

**Introduction:**

Primary transitional cell carcinoma (TCC) of the fallopian tube is an extremely rare tumor.

**Patient concerns:**

A 79-year-old woman presenting with vaginal discharge.

**Diagnosis:**

Pelvic magnetic resonance imaging revealed a predominantly solid mass with a lobulated contour, measuring 5.5 cm × 4.6 cm, in the left ovary. The patient underwent total abdominal hysterectomy with bilateral salpingo-oophorectomy. Pathological analysis revealed a high-grade TCC, measuring 7.5 cm × 4 cm, in the left fallopian tube (International Federation of Gynecology and Obstetrics stage IIB).

**Intervention:**

Forty-three months postoperation, recurrence was diagnosed as peritoneal metastasis. The patient underwent 6 cycles of palliative chemotherapy consisting of cisplatin and gemcitabine, the recommended regimen for TCC of the urinary tract.

**Outcome:**

The patient has survived for 27 months without recurrence after palliative chemotherapy, 76 months after diagnosis.

**Conclusion:**

It is rare that primary TCC of the fallopian tube responds to a urinary tract treatment regimen for TCC, even when followed up for an extended period. More research is warranted to determine which treatment regimen will benefit patients the most.

## 1 Introduction

Primary fallopian tube carcinoma (PFTC) is a rare gynecologic malignancy that accounts for only 0.14% to 1.8% of all gynecological tumors.^[1]^ Preoperative diagnosis of PFTC is extremely difficult because most signs and symptoms are non-specific; therefore, definitive diagnosis of PFTC is largely based on postoperative pathology. The diagnosis of PFTC is usually made on histopathological examination and analysis, with >90% of tumors being papillary serous adenocarcinoma and endometrioid carcinoma.^[1,2]^ Primary transitional cell carcinoma (TCC) of the fallopian tube is an extremely rare tumor. Therefore, there are no guidelines for standard treatment. In general, most patients have been treated using PFTC treatment regimens, and the most commonly used chemotherapeutic agents are carboplatin and paclitaxel. Few patients have experienced relapse, and as such, limited information is available regarding appropriate treatment at the time of relapse.

In the present article, we report a case of TCC in a 79-year-old patient presumably arising from the left fallopian tube, with International Federation of Gynecology and Obstetrics (FIGO) stage IIB. Forty-three months postoperation, she experienced recurrence in the subdiaphragmatic area and peritoneum. After palliative surgery, the patient underwent 6 cycles of cisplatin and gemcitabine palliative therapy, the recommended regimen for TCC of the urinary tract. To date, she has a disease-free prognosis for 27 months.

The characteristics, treatment, and prognosis of this disease are described, along with a review of the relevant literature. Furthermore, we discuss the appropriate palliative chemotherapy for primary TCC of the fallopian tube.

## 2 Case presentation

A 79-year-old, gravida 4, para 4, post-menopausal woman presented with vaginal spotting that began 5 days previously. She was diagnosed with hypertension 10 years previously and treated with calcium channel blockers. She underwent trans-anal excision 1 year previously for rectal adenocarcinoma in situ.

**Figure 1:**
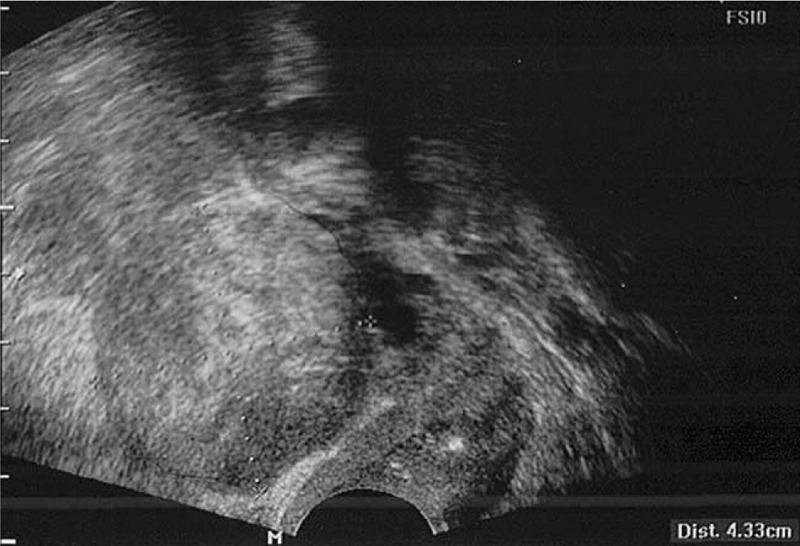
Transvaginal ultrasonography revealing a solid mass, measuring 4.3 cm × 4.3 cm, on the anterior wall of the uterus.

**Figure 2:**
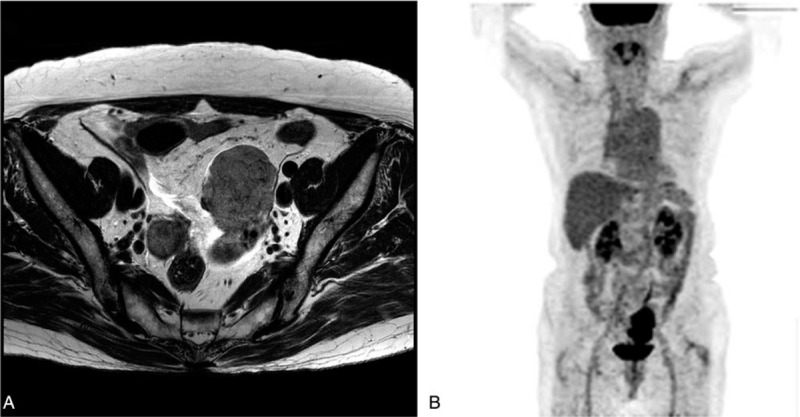
At diagnosis, (A) pelvic magnetic resonance images of a predominantly solid mass, measuring 5.5 cm × 4.6 cm, with a lobulated contour in the left ovary. (B) ^18^Fluoro-2-deoxy-D-glucose positron emission tomography/computed tomography images of a 5.8 cm × 4.2 cm FDG avid mass in the left ovary.

The patient's general condition was good, and vital signs were stable. Physical examination of the abdominopelvic area revealed no palpable mass; vaginal discharge was whitish, mucoid, and scanty. Transvaginal ultrasonography revealed a solid mass on the wall of the anterior uterus measuring 4.3 cm × 4.3 cm; however, it was difficult to determine its origin because both the adnexa and uterus exhibited age-related atrophic changes (Fig. 1). A Papanicolaou (ie, “PAP”) smear revealed reactive cellular changes, and the serum cancer antigen (CA) 125 level was normal (16.80 U/mL [normal, ≤35 U/mL]), whereas other serum tumor markers such as alpha fetoprotein, carcinoembryonic antigen, and CA19-9 were within the normal range. Pelvic magnetic resonance imaging revealed a predominantly solid mass with a lobulated contour measuring 5.5 cm × 4.6 cm in the left ovary (Fig. 2A). ^18^Fluoro-2-deoxy-D-glucose positron emission tomography/computed tomography (CT) revealed an fluorodeoxyglucose (FDG) avid mass, measuring 5.8 cm × 4.2 cm, in the left ovary, with no evidence of renal pelvis, ureter, bladder, or urethral involvement (Fig. 2B).

The patient underwent total abdominal hysterectomy using bilateral salpingo-oophorectomy, pelvic lymphadenectomy, and peritoneal biopsies for suspicion of left ovarian cancer. Both ovaries and the uterus were normal in size and shape; however, the left fallopian tube was ruptured and enlarged, approximating the size of a tennis ball.

**Figure 3:**
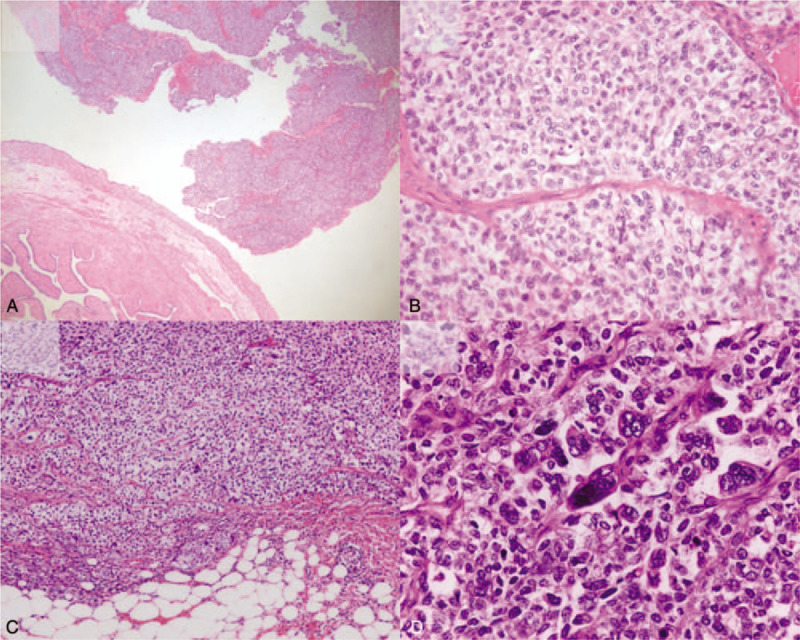
Light microscopy examination. (A) The tumor exhibited a broad papillary growth pattern, which was separated by fibrous septa and composed of solid sheets of cells with distinct borders (hematoxylin–eosin saffron [HES] stain, original magnification × 40). (B) Tumor cells are usually of moderate to large size, and have modest amounts of pale to eosinophilic cytoplasm. The nuclei are oval with clumped chromatin and frequently exhibit mitotic figures (HES stain, original magnification × 400). (C) Recurrent mass exhibiting a nested growth pattern (HES stain, original magnification × 100). (D) Tumor cells exhibit more pleomorphism than primary tumor with occasional tumor giant cells (HES stain, original magnification × 400). FDG = fluorodeoxyglucose, HES = hematoxylin–eosin saffron.

Pathological analysis revealed high-grade TCC (pT2b), measuring 7.5 cm × 4 cm, in the left fallopian tube. The tumor extended beyond the fallopian tube to the pelvic rectal peritoneum. The diagnosis was confirmed as primary TCC (FIGO stage IIB) of the fallopian tube. The tumor consisted of a broad papillary growth pattern with fibrous septa (Fig. 3A). The tumor cells were polygonal and usually of moderate size. The nuclei were oval with clumped chromatin and frequently exhibited mitotic figures (Fig. 3B). Immunohistochemical staining was positive for cytokeratin (CK), CK7, and epithelial membrane antigen and negative for p53, CK20, and p63. The patient underwent 6 cycles of carboplatin (area under the curve, 5) and adjuvant chemotherapy over a 5-month period. ^18^Fluoro-2-deoxy-D-glucose positron emission tomography/CT demonstrated no evidence of FDG-avid recurrent malignancy.

**Figure 4:**
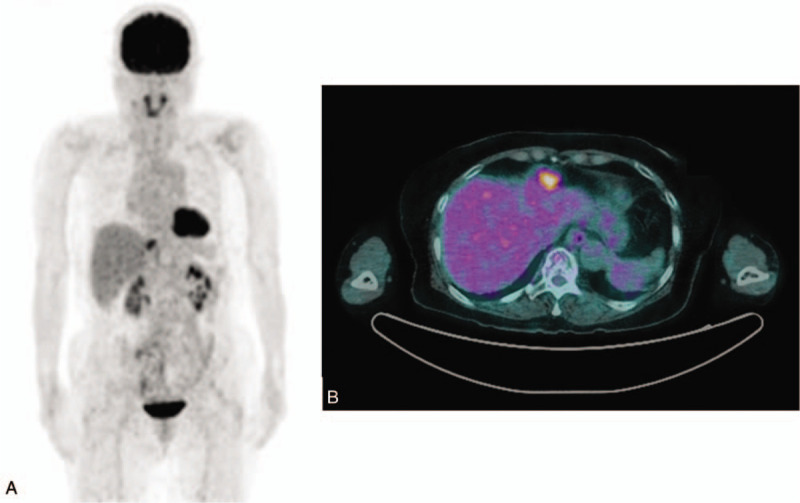
At recurrence (A) and (B) nodular (2.6 cm), markedly intense FDG uptake is apparent in the right sub-diaphragmatic space on positron emission computed tomography/computed tomography.

At 3 years and 7 months postoperatively, a nodular markedly intense FDG uptake, measuring 2.6 cm, was observed in the right subdiaphragmatic space on PET-CT (Fig. 4). Laparoscopic tumorectomy was performed. A 3-cm oval mass was found in the diaphragm above the falciform ligament, and numerous metastatic masses were found in the peritoneum. Histological examination confirmed recurrence of TCC. Tumor cells exhibited more pleomorphism (Fig. 3C ,D). The patient was transferred to the oncology department for palliative chemotherapy.

Gemcitabine (1250 mg/m^2^ on days 1 and 8) and cisplatin (70 mg/m^2^ on day 1) combination therapy, the recommended regimen for TCC of the urinary tract, was administered for 6 cycles as palliative chemotherapy. Considering the advanced age of the patient, the dose was reduced by 30%. The patient tolerated the treatment well, and no grade 3 or 4 toxicity (according to the National Cancer Institute Common Terminology Criteria for Adverse Events version 4.0^[3]^) was observed.

After chemotherapy, complete remission (in accordance with Response Evaluation Criteria in Solid Tumors criteria, version 1.1^[4]^) was confirmed on PET-CT. Chest, abdominal, and pelvic CT was performed on follow-up, and findings of recurrence were not observed for 27 months.

## 3 Discussion

PFTC is the rarest tumor in female genital tract cancers. Serous papillary adenocarcinoma is the most common, and TCC accounts for less than 10%.^[5]^ Primary TCC of the fallopian tube has been reported in a few cases since the initial report by Federman in 1973.^[6]^ Only 10 cases have been reported over a 20-year period.^[5,7–13]^ The clinical features of TCC are summarized in Table 1.

**Table 1:**
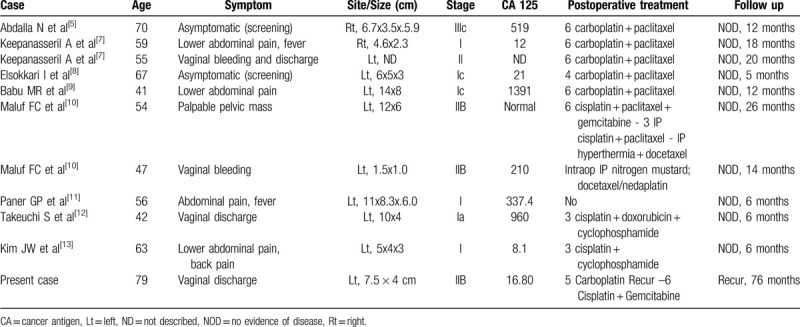
Clinicopathological features of primary transitional cell carcinoma of the fallopian tube.

The origin of TCC of the fallopian tube has been ascribed to transitional cell metaplasia of the tubal serosa or from the mucosal epithelium.^[14]^ PFTC occurs most commonly between 40 and 60 years of age (mean age, 55 years).^[1]^ The median age of patients with TCC of the fallopian tube is 56 years (range, 41–79 years) (Table 1), and it appears to occur at ages similar to those for PFTC. Our patient was the oldest.

Clinically, PFTC is often asymptomatic, and 2 of 11 patients reported in the literature exhibited no symptoms. The most specific symptoms of PFTC were vaginal bleeding and discharge, abdominal pain, and pelvic mass. In 15% patients, Latzko's triad was reported, consisting of vaginal discharge, colicky abdominal pain, and pelvic mass.^[1,2]^ In TCC, the major symptom was vaginal discharge or bleeding (4/11), pelvic pain (4/11), and pelvic mass (1/11); however, no patient exhibited all of Latzko's triad. However, the low incidence rate makes it difficult to draw conclusions from these results alone.

CA 125 levels are elevated in more than 80% of PFTC cases, especially in those with advanced disease; it is also a useful tool for monitoring disease progression.^[1,2,8]^ However, CA 125 level in TCC was elevated in 50% (5/10) patients reported in the literature. In our case, the levels in the patient were within normal limits. The clinical value of CA 125 in TCC is believed to be inferior to those in PFTC.

PFTC is often preoperatively misdiagnosed as ovarian malignancy.^[1]^ In TCC, most cases did not predict preoperative fallopian tube origin, and 1 case was suspected of cervical cancer.^[7]^ In PFTC, mutation in *BRCA* 1,2 is a known risk factor.^[15]^ In 1 case, the *BRCA* 1,2 test was performed, but the result was negative; further study is needed to confirm any association(s).^[8]^

Surgery is the preferred treatment for PFTC and is similar to the treatment for ovarian cancer—cytoreductive surgery with removal of as much of the tumor as possible. The procedure of choice is total abdominal hysterectomy, bilateral salpingo-oophorectomy, selective pelvic and para-aortic lymphadenectomy, peritoneal washing, and peritoneal biopsies.^[1,2,15]^ PFTC usually requires adjuvant chemotherapy owing to the high risk of recurrence after surgical resection irrespective of a successful operation. There is no consensus regarding the role of adjuvant radiotherapy. Because PFTC is mostly adenocarcinoma, and histologically similar to epithelial ovarian cancer, combination therapy with carboplatin and paclitaxel is the standard therapy.^[1,2,15]^ In TCC, 5 of 11 patients reported in the literature received carboplatin and paclitaxel combination chemotherapy.

As a paradigm in medical oncology, treatments are directed according to specific histological types instead of the primary site of tumor origin.^[10]^ However, owing to its rarity, no firm recommendations can be made based on the scant data available for TCC of the gynecologic tract. TCC of the urinary tract is known to be a chemo-sensitive tumor.^[10]^ Treatment of unresectable or metastatic TCC of the urothelium includes the platinum-based chemotherapy. Cisplatin and gemcitabine are considered standard regimen owing to a more favorable toxicity profile.^[10,16]^

In our case, the patient received carboplatin as adjuvant chemotherapy. Recurrence of primary TCC of the fallopian tube was diagnosed as peritoneal metastasis. We recommended treatment with cisplatin and gemcitabine, the standard therapy for TCC of the urinary tract. Palliative chemotherapy was administered for six cycles, and she has survived 27 months without recurrence. Among published cases of TCC, relapse occurred only in our patient. There are few, if any, reports describing the effects of chemotherapy on the relapse of TCC of the fallopian tube.

Primary TCC of the fallopian tube is rarely reported; as such, it is difficult to clearly explain how histological features affect prognosis. Uehira et al analyzed 21 cases of PFTC, including 12 cases of TCC with a predominantly histological pattern (>50%). The authors reported that TCC-predominant tumors recurred later (mean, 31.2 months versus 14.4 months after diagnosis) than non-TCC-predominant tumors, resulting in a significant difference in the 2-year disease-free survival rate.^[17]^ This report suggests better prognosis for primary TCC of the fallopian tube than for other subtypes such as serous adenocarcinoma.

Primary TCC of the fallopian tube is rare and has no standardized treatment guidelines. We reported a rare case of primary TCC of the fallopian tube in a 79-year-old woman with peritoneal metastasis 43 months after surgery. To date, she has a disease-free prognosis 27 months after palliative chemotherapy for TCC of the urinary tract. Our case represents a therapeutic limites as the basis for the current treatment recommendation in that most fallopian tube tumors are serous and thus should be treated as ovarian cancers. Whether this applies to TCC that more closely resembles urinary tract carcinoma is questionable. More studies are required to develop more appropriate treatment regimens for patients with primary TCC of the fallopian tube.

## 4 Conclusion

Primary TCC of the fallopian tube is a rare, malignant tumor of the female reproductive tract. Similar to PFTC, the most common symptoms of TCC are vaginal discharge, abdominal pain, and pelvic mass. Moreover, the median age at occurrence is also similar (56 years). Preoperative diagnosis is extremely difficult, and definitive diagnosis is largely based on postoperative pathological findings. For patients who undergo upfront cytoreductive surgery, adjuvant chemotherapy includes a platinum doublet, traditionally carboplatin and paclitaxel. However, it is difficult to conclude whether this regimen is appropriate for TCC similar to urinary tract carcinoma. We reported a case of a 79-year-old patient with TCC arising from the left fallopian tube, FIGO stage IIB. Forty-three months postoperatively, the disease recurred in the peritoneum. After palliative surgery, cisplatin and gemcitabine palliative chemotherapy, the recommended regimen for TCC of the urinary tract, was administered. To date, she has a disease-free prognosis for 27 months. In this study, we performed a long-term follow-up of 6 years, and we have demonstrated a good response to the standard treatment for TCC of the urinary tract in a patient with primary TCC of the fallopian tube. Further studies, however, will be needed to establish a standard of care for TCC of the fallopian tube.

## Acknowledgment

We would like to thank Editage (www.editage.co.kr) for English language editing.

## Author contributions

**Conceptualization:** Dong-Hyu Cho, Chang-Yeol Yim MD, Na-Ri Lee.

**Data curation:** Chang-Yeol Yim MD.

**Methodology:** Kyoung Min Kim.

**Supervision:** Dong-Hyu Cho, Chang-Yeol Yim MD.

**Writing – original draft:** Dong-Hyun Lee.

**Writing – review & editing:** Na-Ri Lee.
